# Relative Abundance of Alpha-Amylase/Trypsin Inhibitors in Selected Sorghum Cultivars

**DOI:** 10.3390/molecules25245982

**Published:** 2020-12-17

**Authors:** Sorel Tchewonpi Sagu, Eva Landgräber, Michal Rackiewicz, Gerd Huschek, Harshadrai Rawel

**Affiliations:** 1 Institute of Nutritional Science, University of Potsdam, Arthur-Scheunert-Allee 114-116, 14558 Nuthetal, OT Bergholz-Rehbrücke, Germany; sorelsagu@uni-potsdam.de (S.T.S.); e.landgraeber@live.de (E.L.); rackiewicz@uni-potsdam.de (M.R.); 2 IGV-Institut für Getreideverarbeitung GmbH, Arthur-Scheunert-Allee 40/41, 14558 Nuthetal, OT Bergholz-Rehbrücke, Germany; gerd.huschek@igv-gmbh.de

**Keywords:** sorghum, α-amylase/trypsin inhibitors, reducing agents, cysteine alkylation, SDS PAGE, targeted proteomics, LC–MS/MS

## Abstract

Sorghum is of growing interest and considered as a safe food for wheat related disorders. Besides the gluten, α-amylase/trypsin-inhibitors (ATIs) have been identified as probable candidates for these disorders. Several studies focused on wheat-ATIs although there is still a lack of data referring to the relative abundance of sorghum-ATIs. The objective of this work was therefore to contribute to the characterization of sorghum ATI profiles by targeted proteomics tools. Fifteen sorghum cultivars from different regions were investigated with raw proteins ranging from 7.9 to 17.0 g/100 g. Ammonium bicarbonate buffer in combination with urea was applied for protein extraction, with concentration from 0.588 ± 0.047 to 4.140 ± 0.066 mg/mL. Corresponding electrophoresis data showed different protein profiles. UniProtKB data base research reveals two sorghum ATIs, P81367 and P81368; both reviewed and a targeted LC–MS/MS method was developed to analyze these. Quantifier peptides ELAAVPSR (P81367) and TYMVR (P81368) were identified and retained as biomarkers for relative quantification. Different reducing and alkylating agents were assessed and combination of tris (2 carboxyethyl) phosphine/iodoacetamide gave the best response. Linearity was demonstrated for the quantifier peptides with standard recovery between 92.2 and 107.6%. Nine sorghum cultivars presented up to 60 times lower ATI contents as compared to wheat samples. This data suggests that sorghum can effectively be considered as a good alternative to wheat.

## 1. Introduction

Sorghum is a cereal mainly growing in arid and semi-arid regions representing the fifth most important globally produced cereal [1,2]. It is traditionally used as an animal feed constituent in many Western countries but more importantly it serves as a staple food for a large population in many tropical and subtropical regions [3,4,5]. The demand for gluten-free products has grown in recent years due to the increasing number of people suffering from allergies related to wheat-based products [6]. The so-called gluten-free cereals are attracting more interest and sorghum, especially the species *Sorghum bicolor* (L.) Moench, is one of the most promising candidate identified in this context [7]. In fact, sorghum has been reported to be a safe alternative for people suffering from celiac disease and to be an important source of bioactive components such as phenolic acids and flavonoids [3,8]. Innovative processing of sorghum seeds leading to the development of new products in order to promote its use either as a flour mixture with wheat or as pure flour has also been recently discussed as favorable options [6,9,10].

Oria et al. [11] reported that consumers of sorghum-based diets in Africa, Asia and South America were highly dependent on the protein and energy available in cereals. Since the quantity and protein composition of cereals is a determining factor in assessing the technological and nutritional qualities of cereals, many studies have also been focused on sorghum proteins in this context. Glutelin fraction was initially reported accounting for more than half of the total protein content of sorghum and that only less than half of the total protein fractions could be accounted to extractable protein nitrogen as determined by the Kjedahl assay [12]. Prolamins, also termed kafirins, represents around 80% of the sorghum proteins, allocated correspondingly to storage proteins [13]. The most well-known kafirins are α-kafirins, which account for 80–84% of the prolamines in the seeds [14]. The kafirin α-helical structures are rich in hydrophobic amino acids capped with glutamine rich ends. The other kafirins types, β-, δ- and γ-kafirins, are particularly rich in cysteine and methionine residues [15]. Aqueous alcohol with reducing agent and basic buffer containing sodium dodecyl sulfate has been generally used to extract these protein fractions [16,17]. Besides kafirins, other small sorghum protein fractions have also been identified, among them α-amylase/trypsin inhibitors (ATIs) [18].

ATIs, with bifunctional activity against both α-amylase and trypsin, have been identified and isolated from various plant and grains species. In plants, ATIs serve as a defense mechanism against pests by influencing the activity of enzymes important for the digestion of carbohydrates and proteins. They are small molecules, with molecular weights often between 10 and 16 kDa, containing in their structures 8–10% of cysteine residues, which can constitute correspondingly 4–5 disulfide bonds [19]. ATIs in wheat have been found to associate themselves to dimer or tetramer structures [20]. To our knowledge, no such associations have been reported for sorghum ATIs. They exhibit strong sequence similarities with the purothionins, a wheat allergen associated with severe allergy [17,21]. As of 15 November 2019, the online UniProt Knowledgebase (UniProtKB) contained two different entries for sorghum ATIs: proteins P81367 and P81368; both reviewed.

ATIs have been mentioned as being responsible for a type of allergy related to wheat consumption, also called non-celiac wheat sensitivity (NCWS). It is known that the risk of developing an allergy depends on the level/concentration of these molecules in a cereal [20]. Although extensive studies referring to the characterization of wheat ATIs and their related toxicity have been recently addressed, nevertheless, relatively little information about characteristics of sorghum ATIs, their relative proportion and their potential to induce such disorders are known. Most of the available work refers to biological studies of sorghum ATIs [8,22] and their preliminary characterization [23]. Studies investigating the non-toxicity of sorghum using either in vitro or in vivo assays have also been reported [2,24,25,26,27,28]. Bokka et al. [29] claimed the possible allergenicity of sorghum pollen grains (*Sorghum bicolor Polcalcin* species) as panallergen. They attempted an in silico modeling and simulation studies in order to characterize the antigenic determinants of this sorghum species responsible for eliciting the IgE response, but the specificity of this allergen is still yet not completely characterized and documented.

Recent studies and our own work indicate that selected peptides originating from different proteins can be identified, being specific to these proteins and may be used to assess the corresponding proteins on a quantification scale [20,30,31,32]. The strategy involves different steps: selection of marker proteins based on the thorough research of literature and databases, in silico digestion with different proteolytic enzymes, potential matches to test for the specificity of the peptides liberated and to encompass their response by a multiple reaction monitoring (MRM) approach for chromatography coupled with tandem mass spectrometry (LC–MS/MS). Finally, the whole workflow has to be optimized to address the matrix related effects and is also generally termed as a targeted proteomic approach [32].

Considering the current growing interest in sorghum, and to support the cultivation and consumption, it would therefore be valuable to improve knowledge about the content of ATIs from different sorghum cultivars, especially while considering the potentials elucidated above. Their quantification and better characterization is therefore an essential step in this process. The objective of the present work was to contribute to the characterization and relative quantification of sorghum ATIs using targeted proteomics tools. Fifteen sorghum cultivars from different regions were analyzed and two wheat samples were used to “relatively compare” their ATI contents. The ammonium bicarbonate buffer in combination with urea was applied for protein extraction and proteomics grade trypsin was used to perform the digestion. The ATIs in sorghum were subjected to different reducing and alkylating agents to compare and determine the best conditions for the subsequent analysis in the established workflow. Finally, an optimized LC–MS/MS method was developed in order to analyze ATIs in sorghum cultivar.

## 2. Results and Discussion

### 2.1. Protein Concentration and SDS PAGE

The Lowry method [33] was used to determine the protein concentration of Ambi/urea extracts prior to their digestion and the results are showed in Table 1. Sorghum samples were found to express various concentrations of protein. Significant higher values of extracted protein content were found in the American cultivar SOR 777 (4.046 ± 0.106 mg/mL) and Asian cultivar SOR 618 (4.140 ± 0.066 mg/mL). Protein concentrations ranged between 2.049 ± 0.026 and 3.719 ± 0.238 mg/mL for the other samples. The exception was cultivar SOR 844 from Asia, which yielded a very low protein concentration (0.588 ± 0.047 mg/mL) compared to other cultivars. This is contrasted by the results of the total protein content according to Kjeldahl method. Cultivar SOR 844 delivered a total protein content of 12.1 g/100 g that was the average of the other samples. However, samples B.Sc and R.Sc from Cameroon showed the lowest total protein levels (7.9 and 8.5 g/100 g, respectively), while cultivars SOR 1055 (15.4 g/100 g) and SOR 1099 (17.0 g/100 g) exhibited the highest protein values. These observed differences between the trends of the crude proteins and those extracted highlights the significant contrast in protein composition of each cultivar, and therefore their ability to be more or less easily extracted by the extraction buffer combination of Ambi/urea. According to the nature and composition of kafirin (77–82% of total protein), sorghum proteins have been readily extracted with alcohol and/or a reducing agent such as β-mercaptoethanol to improve the yield [14,15].

To compare the composition of the extracted proteins, SDS-PAGE was performed under denaturing and reducing conditions and the results are presented in Figure S1 (Supplementary Materials). For the most of the samples, the trend of the band intensities corroborated the results of the Lowry assay. Protein bands were also found in the range of the molecular weights generally allocated to ATIs (10–15 kDa).

### 2.2. Establishment of the LC–MS/MS Method for the Analysis of Sorghum ATIs: Selection of Peptides

Sorghum cultivars SOR 958 and SOR 1236 were initially used to assess the selection of peptides. From the in silico digestion using Skyline software (version. 20.2) [34], and according to the set peptide parameters, a total of 9 and 8 peptides containing 4–25 amino acids were preselected for proteins P81367 and P81368, respectively. Six or more transitions and fragments of each peptide were initially assessed. Table S1 (see Supplementary Materials) presents the different peptides with their analytical characteristics. After optimizing the retention times and collision energies, a biomarker peptide (quantifier peptide) was selected for each of the two sorghum ATIs. The selection was made on the basis of the three main criteria: (1) the specificity of the peptides (uniqueness tested through the “unique peptide” function of the Skyline software, using the sorghum proteome obtained from the Uniprot online database as the background); (2) the peptide signal intensity and consistency and (3) peptides containing cysteine residues were excluded (this was done to avoid modifications related to cysteine residues due to the alkylation performed during sample preparation). Peptides ELAAVPSR (*m/z*: 421.7; charge state 2; with fragments A [y6], A [y5], V [y4] and P [y3]) and TYMVR (*m/z*: 335.1731; charge state 2; with fragments Y [y4], M [y3] and Y [b2]) were identified and retained as biomarkers for ATIs P81367 and P81368, respectively. Their corresponding best retention time/optimal collision energies were 8.59 min/14.1 eV and 8.21 min/8.4 eV for peptides ELAAVPSR and TYMVR, respectively. No data referring to sorghum ATIs biomarkers are actually available in the literature.

In addition to the biomarkers, two qualifier peptides were also retained in the final method in order to improve the identification and significance of the protein analysis. Peptides IYAVSR and TCGLGGPYGPVDPSPVLK; and LVPY**C**R and TLHGRPF**C**YALGAEGTTT were accordingly selected as qualifier peptides for protein P81367 and P81368, respectively. The amino acid cysteine (C) is marked on purpose to indicate that it was modified during the sample preparation.

### 2.3. Improvement of the Trypsin Digestion: Evaluation of Different Reducing and Alkylating Agents

**Figure 1:**
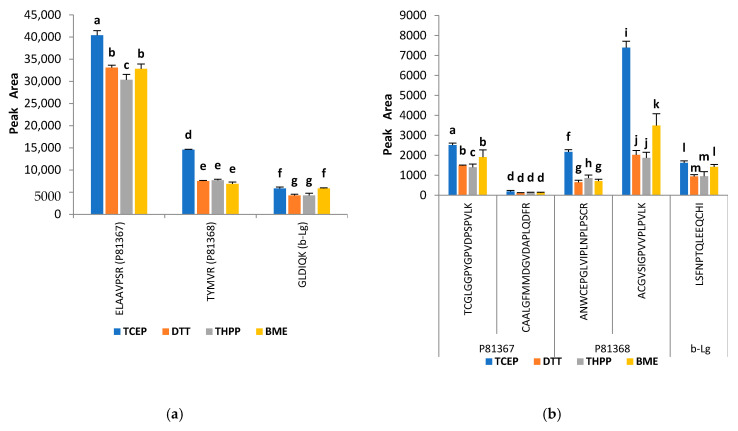
Effect of different reducing agents on (**a**) quantifier peptides and (**b**) cystine-containing peptides of ATIs P81367, P81368 and β-lactoglobulin (β-Lg). Iodoacetamide was used as alkylating agent. Reducing agents: TCEP-Tris (2 carboxyethyl) phosphine; DTT-dithiothreitol; THPP-Tris(hydroxypropyl)phosphine; BME-beta-mercaptoethanol. Data are expressed as means ± standard deviation, *n* = 3. Comparisons were performed per family and different letters indicate significantly different values (*p* < 0.05).

**Figure 2:**
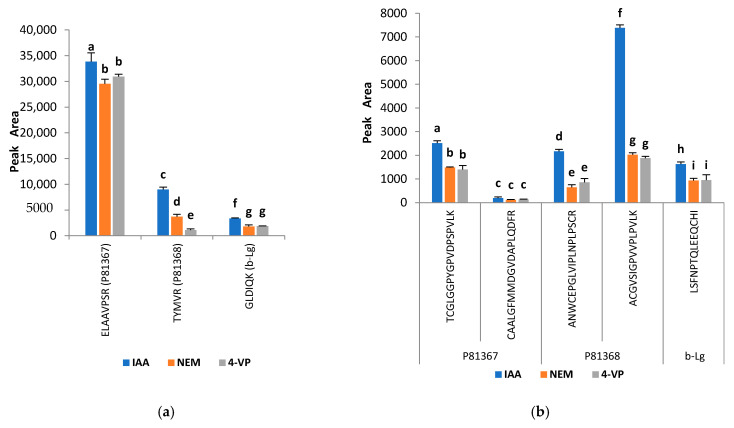
Effect of different alkylating agents on (**a**) quantifier peptides and (**b**) cystine-containing peptides of ATIs P81367, P81368 and β-lactoglobulin (β-Lg). Tris (2 carboxyethyl) phosphine (TCEP) was used as a reducing agent. Alkylating agents: IAA—iodoacetamide; NEM—*N*-ethylmaleimide and 4-VP—4-vinylpyridine. Data are expressed as means ± standard deviation, *n* = 3. Comparisons were performed per family and different letters indicate significantly different values (*p* < 0.05).

It is well known that ATI molecules contain in their structure 8–10% cysteine residues, which are known to form four disulfide bonds via the thiol groups [35,36]. This leads to the development of stable and structural rigid protein complexes. The consequent interaction of the ATI molecules may further occur as reported for wheat ATIs [35,37,38], although this process has not yet been specifically reported for sorghum species. In order to allow complete enzymatic digestion to produce a full spectrum of peptides (analytes), efficient reduction of disulfide bonds followed by blocking of the reactive -SH sites are of key importance. To this end, we tested four different reducing (tris(2 carboxyethyl)phosphine-TCEP, dithiothreitol-DTT, tris(hydroxypropyl)phosphine-THPP and β-mercaptoethanol-BME) and three alkylating agents (iodoacetamide-IAA, *N*-ethylmaleimide-NEM and 4-vinylpyridine-4VP). Initially, the conventional alkylating agent IAA was first used to alkylate the samples while applying the different reducing agents. The analyses were carried out in triplicate and the total peak areas of quantifier and cysteine-containing peptides were compared. The results are presented in Figure 1 and Figure 2. When comparing the efficiency of the four reducing agents, it is evident that TCEP led to an increase in the intensity of the detected peptides; this applies both for the quantifier and for cysteine-containing peptides. All analyzed peptides exhibited higher responses with TCEP and the statistical analysis showed that they were significantly different (*p* < 0.001) from the responses of the three other reducing agents for quantifier peptides ELAAVPSR and TYMVR (Figure 1a), and for cysteine-containing peptides TCGLGGPYGPVDPSPVLK, ANWCEPGLVIPLNPLPSCR and ACGVSIGPVVPLPVLK (Figure 1b). The other analyzed peptides (qualifier peptides) showed the same trend (Figure S2, see Supplementary Materials). Several works have been carried out on this subject with diverging results. For example, Rogers et al. [39] in their study on protein thiol modifications found DTT and TCEP to be equally effective in reducing mixed disulfides bonds. Systematical evaluation of the commonly reduction reagents DTT, BME, TCEP and THPP has also been formerly addressed and the number of peptides identified thereof were very similar for the mentioned reducing reagents [40]. The results with TCEP confirm these observations and was then selected and used in the protocol as a reducing agent during the sample preparation.

To evaluate the different alkylating agents while now using TCEP as the common reducing agent, it was possible to evaluate the response of the analyzed peptides and to compare them (Figure 2). The outcome is clear, showing that IAA produced among the tested options the best results. All peptides showed significantly different peak area values with this alkylating agent, and the responses were even more than 2-fold higher with IAA as compared to the applied reagents NEM and 4VP for the peptides TYMVR, TCGLGGPYGPVDPSPVLK, ANWCEPGLVIPLNPLPSCR and ACGVSIGPVVPLPVLK. Lower peak area values were measured from samples alkylated with NEM and 4VP and in some cases; the responses obtained for both chemicals appeared to be similar. The results obtained for the other analyzed peptides again reflected the similarity in observations discussed here (Figure S3, Supplementary Materials). The same trend was observed for β-lactoglobulin added to the sorghum samples to monitor the digestion progress as documented in Figure 2. These observations do differ from those reported in the literature. On one side, Sebastiano et al. [41] showed that 4VP was able to react and selectively alkylate -SH groups in proteins, achieving up to 100% alkylation of all -SH residues even in complex proteins in contrast to IAA. On the other side, Hill et al. [42] indicated that the reaction of NEM with thiols was predicated to be more effective. In a study comparing three commonly alkylating agents, NEM yielded higher efficiencies and alkylation rates than either iodoacetamide or iodoacetic acid at pH 4–8, at low concentrations and in a shorter period of time [39]. While comparing IAA, acrylamide, NEM and 4VP, the highest number of peptides with IAA-alkylated cysteine has also been reported [40]. However, it should be pointed out that IAA is commonly used for protein thiol alkylation and in view of our results; IAA was finally selected and used in the subsequent steps of this work.

### 2.4. Matrix Effects and Ion Suppression

Matrix effects on analytes are well known in analytical techniques, and this is particularly pronounced with LC–MS/MS methods. During the analysis, the alteration of the ionization efficiency of the target analytes in the presence of coeluting compounds from the matrix (sample solutions) generally leads to a loss of response (ion suppression) [43,44].

For the assessment of matrix effects in quantitative analysis of sorghum ATIs with the developed method, β-lactoglobulin and an internal standard were used at different stages of the sample preparation. β-lactoglobulin was introduced into the sample extracts before starting digestion. Reduction, alkylation, tryptic digestion and sample cleaning (SPE) steps were thus carried out in the presence of β-lactoglobulin. A blank consisting of distilled water instead of sample extract was also constituted in order to serve as a control. This process allowed one to follow-up the matrix effect and its impact particularly on tryptic digestion.

**Figure 3:**
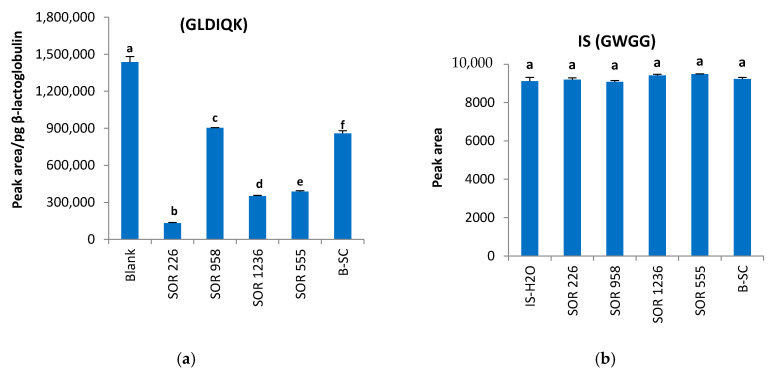
Effects of the matrix of sorghum cultivars SOR 226, 958, 1236, 555 and B.Sc on (**a**) β-lactoglobulin and (**b**) internal standard. Data are expressed as means ± standard deviation, *n* = 3. Different letters indicate significantly different values (*p* < 0.05).

On the other hand, a short peptide consisting of four amino acid residues (GWGG) was used as an internal standard and introduced into the samples after the SPE process and just before the LC–MS/MS analysis. In this context, the effect of the matrix components on the analytes during the LC–MS analysis was assessed (obstruction/interference with the analytes to gain charge during ionization). All these tests were carried out on five different sorghum cultivars and the results are presented in Figure 3. Due to the high cost of pure synthetic peptides and since at the beginning of this work there was no indication in the literature about potential biomarkers of sorghum ATIs P81367 and P81368, this procedure was initiated to initially develop the LC–MS/MS method. Thus, performing a relative quantification allows having a first overview on the level of ATIs contained in the different sorghum samples. Once the method is established and validated and the biomarkers identified, it will thereafter be possible in a later phase to acquire synthetic isotope labeled peptides identical to the biomarkers in order to conduct absolute quantification analysis.

It appeared that lower β-lactoglobulin values were detectable by mass spectrometry in all samples compared to the control (Figure 3a). Analysis of variance indicated that these β-lactoglobulin concentrations in the samples were all significantly different from the control (*p* < 0.001). However, losses were not identical in all samples. The behavior of the sorghum cultivar SOR 226 is particularly noteworthy. It exhibited 91% less intensity of the β-lactoglobulin quantifier peptide (GLDIQK), while losses in SOR 958 and B.Sc samples were 37% and 40%, respectively. Conversely, the effect of the matrix impacted differently the analysis of the internal standard. Signal losses/gains of the internal standard were all within 4% (Figure 3b), and the differences compared to the blank appeared not to be significantly different.

Although it would have been ideal to have isotopic peptides of the selected biomarkers, the retention time of the IS GWGG (8.7 min) and the β-lactoglobulin peptide GLDIQK (8.5 min) being similar to those of peptides ELAAVPSR (8.6 min) and TYMVR (8.2 min) biomarkers suggested that they may be subject to similar matrix effects. Considering these results, at least two hypotheses could be formulated: (1) the matrices of the different sorghum cultivars exerted a significant effect on β-lactoglobulin during the digestion process making it less accessible to trypsin and (2) the SPE step permitted to clean the sample solutions separating the analytes from the other matrix compounds, thus allowing a smaller impact of the matrix effect on the analysis (lower losses of the internal standard signal). Thus, in the further work, we opted to maintain the same procedure using on one hand β-lactoglobulin to monitor the regularity of the digestion process, and on the other hand, to use the ratio between the intensities of the internal standard in the water and in the samples to normalize the values of the peak areas of the ATI biomarkers.

### 2.5. Method Validation

Method validation is a key step in the analytical method development process prior to the measurements. For that purpose different validation criterion were investigated, including the internal standard recovery in samples, the repeatability, the reproducibility, the linearity and the limit of detection and limit of quantification. Data analysis was performed with the Excel Analysis Package (version 2010) and GraphPad Prism version 8.0.0 for Windows (GraphPad Software, San Diego, CA, USA) and a summary of the results is presented in Table 2.

One of the major factors affecting the performance of MS analysis is the recovery of the internal standard that may be affected by other compounds in the samples (the matrix effect). In order to minimize this matrix effect, we opted to introduce the internal standard in the samples after the SPE step and just before starting the measurements. Five samples were used to evaluate the recovery of the internal standard. Four different experiments were performed at different days. Each experiment was carried out in triplicate and the results are shown in Figure S4 (Supplementary Materials). The recovery of the internal standard was found to be between 92.2 and 107.6%. A two-way ANOVA followed by Turkey multiple comparisons test was performed using GraphPad Prism with a family-wise significance and confidence level of 0.1 (90% confidence interval). The results showed that the interactions were statistically not significant.

Supplementary Figure S5a,b present the results of the intra- and inter-day analysis of ATIs P81367 and P81368 using sorghum samples SOR 226, 958, 1236, 555 and B.Sc. The relative standard deviation (% RSD) was used to evaluate repeatability and reproducibility. It indicates whether the standard deviation is a small or a large amount relative to the mean of the data. In general, the results showed a relative standard deviation in the range of 0.12–2.3% of the means for the intraday analysis, and 1.42–5.21% of the means for the interday analysis; indicating that the data were closely approximate to the means. The RSD thus demonstrated good precision of the developed LC–MS/MS method and confirmed the good repeatability and reproducibility of the results.

Linearity was assessed by injecting into the HPLC system six different amounts of the internal standard (10–200 pg), β-lactoglobulin (2–40 ng) and Wray and Tech 8 digested solutions (16–320 ng equivalent protein according to the Lowry method). Each solution was injected in triplicate and a linear regression analysis was performed. The results showed that the internal standard and β-lactoglobulin and proteins P81367 and P81368 were all linear within the selected ranges of variation (Figure S6, see Supplementary Materials). The coefficients of determination ($R^{2}$) were all between 0.9892 and 0.9999. More interestingly, it is observed that the slopes of the internal standard (IS) in water (57.9) and those of IS in samples SOR 1236 (63.9) and SOR 958 (60.1) exhibited similar values (Figure S6a). The analysis of variance indicated that there were no significant differences between them. On the other hand, the situation was different with β-lactoglobulin. A higher value of the slope was found for the blank (1725.4) while it was only half in the samples, with 952.7 and 871.2 in Wray and Tech 8 samples, respectively (Figure S6b, Supplementary Materials). This result highlights once again the important effect of the sample matrix on the analysis for β-lactoglobulin in a complex protein mixture.

The linear regression method (y=a + bx) was used to determine the limit of detection $\left(LoD=3SD_{a}/b\right)$ and the limit of quantification $\left(LoQ=10SD_{a}/b\right)$ according to the International Conference on Harmonization (ICH); with $SD_{a}$ the standard deviation of intercept and b, the slope of the regression curve. The regression equations from the linearity of internal standard in water, blank and sorghum cultivar SOR 958 were employed and the results are presented in the Table 1. The results revealed that a minimum amount of 1.155 ng protein was required to perform a quantitative analysis of both P81367 and P81368 ATIs from sorghum samples using the developed method.

### 2.6. Relative Quantification of ATIs from the Fifteen Sorghum Samples

**Figure 4:**
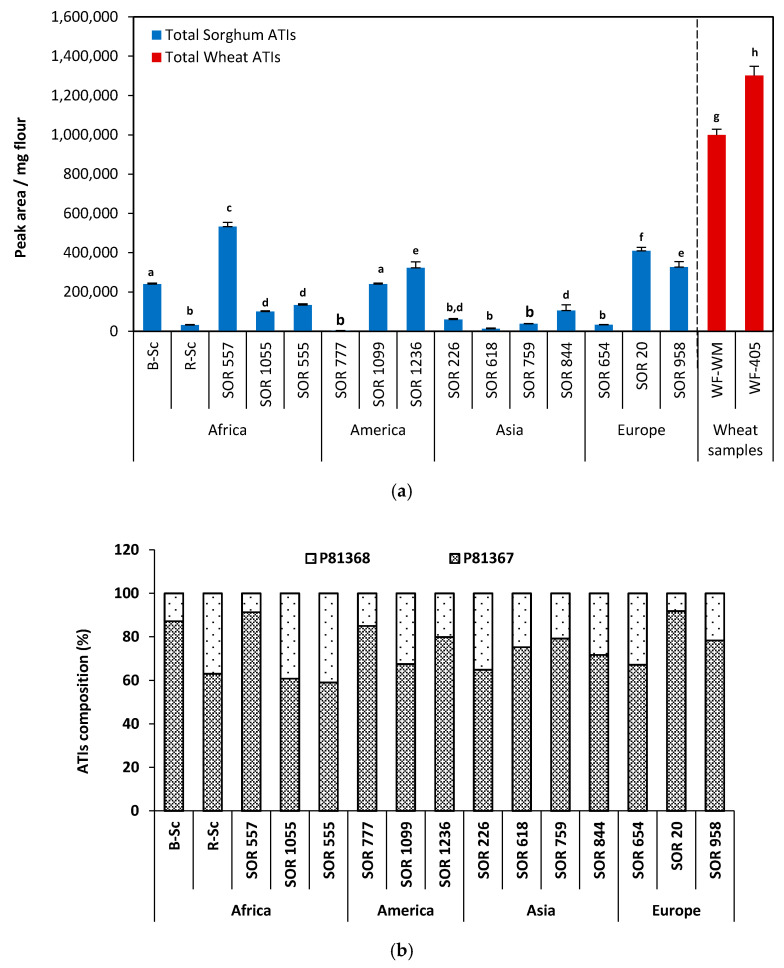
Comparison of (**a**) relative ATIs content of sorghum samples expressed in peak area/mg flour and (**b**) proteins P81367 and P81368 distribution. Data are expressed as means ± standard deviation, *n* = 3. Different letters indicate significantly different values (*p* < 0.05).

Figure 4 shows the ATI content of 15 sorghum cultivars from different regions determined using the developed LC–MS/MS method. The two selected biomarkers ELAAVPSR (P81367) and TYMVR (P81368) were used to evaluate the relative quantification. Three replicate analyses were performed and the internal standard was used each time to normalize the peak areas. The sum of both proteins was calculated and the results were expressed in terms of total peak area of ATIs per mg sorghum flour. Significant differences in relative ATI content were found in the sorghum samples.

Among the cultivars from Africa, SOR 557 exhibited the highest ATIs content (532,779 ± 21,343 PA/mg flour) while R.Sc showed only 31,764 ± 1875 PA/mg flour. With values of 100,111 ± 3747 and 133,317 ± 6489 PA/mg flour, SOR 1055 and SOR 555 yielded similar ATI contents and did not differ significantly from each other (*p* > 0.05). Similar trends were obtained with cultivars from America and Europe. For example, the American samples SOR 1099 and SOR 1236 yielded ATIs content of 240,494 ± 4634 and 322,678 ± 20,617 PA/mg flour, respectively, while SOR 777 showed only a value of 3845 PA/mg flour. From European cultivars, samples SOR 20 and SOR 958 showed high ATIs content (with 410,002 ± 16,961 and 326,784 ± 27,360 PA/mg flour, respectively) compared to cultivar SOR 654 (32,412 ± 1325 PA/mg flour).

However, generally remarkably low levels of ATIs were observed in sorghum cultivars from Asia. The sorghum extracts obtained from all samples tended to have lower ATIs contents than the other samples: 106,063 ± 8412; 59,329 ± 2164; 37,766 ± 2176 and only 12,227 ± 603 PA/mg for SOR 844, SOR 226, SOR 759 and SOR 618, respectively (Figure 4a). Comparing to other regions, cultivars from Asia contained about 4 times less ATIs (on average). Finally, with the exception of the samples from Asia with low levels of ATIs, no clear trend in term of ATI contents and region of origin of the samples can be observed. The analyzed sorghum samples may differ according to their growing regions and fluctuations within the region are also possible. The R.Sc. sorghum cultivar from Cameroon debited one of the lowest values, while B.Sc. from the same country showed approximately 10 times more ATIs (Figure 4a).

The significant differences observed in terms of ATI content could raise the questions of the cultivation patterns in the different growing regions. In fact, before being domesticated and produced industrially throughout the world for human consumption, sorghum was initially grown in dry regions; including tropical, subtropical and temperate regions between latitudes 45° N and 45° S [45]. In addition, Berenguer and Faci [46] showed that differential treatments and plant densities significantly affected the grain yield of sorghum but also its physicochemical parameters. Finally, besides cultivation regions, the genetic predisposition also seems to prevail. For example, SOR 777 from Mexico, the unique sorghum cultivar from the family *S. halepense* exhibited the lowest amount of ATIs while comparing it to all the other cultivars (from *Sorghum bicolor* species). No data referring to the quantification of ATIs in different species has been described in the literature so far. Sorghum is classified into three main families: *S. bicolor* and two rhizomatous taxa, *S. halepense* and *S. propinquum* [47,48]. *S. bicolor* represents all annual cultivated sorghum while *S. halepense*, considered as a perennial weed is not intensively cultivated because it is both time consuming and difficult [47].

Comparing the composition in both proteins, it appeared that P81367 was the predominant ATIs in all sorghum cultivars. It represented about 59–92% of the total ATI content (Figure 4b). Especially in the SOR 20 sample, this protein accounted for about 91.8% of the total content, while P81368 represented only about 8%. In the sorghum samples SOR 1055 and SOR 555, the ATI contents were composed of approximately equal proportions of both proteins. P81367 represented about 57% and P81368 about 43% of the total relative ATI contents (Figure 4b).

The low level of ATIs in SOR 777 sample is of particular interest and it would then be interesting to analyze more cultivars of *S. halepense* species. It could then serve in further popularizing this sorghum species as ATIs-free candidate accessible for people with cereal related sensitivity disorders as also discussed further below. Unfortunately in this context, the corresponding immune-toxicological response has not yet been fully documented for sorghum cultivars and still needs to be addressed.

The role of wheat ATIs related to the NCWS, also classified as allergenic component for inducing baker’s asthma is now well discussed [32,37,49,50]. Since the biological role of ATIs is the same, it would still be important to know whether the amounts of ATIs detected in sorghum cultivars are sufficient to trigger this immune-toxicological response. Relative high content of sorghum ATIs similar to those of wheat could lead to the similar disorders, while relative low amounts could make sorghum a good alternative to wheat for people suffering from them. Finally, we opted to present a short comparison of the two cereals, sorghum and wheat, keeping in mind that only relative contents are available and that the comparison would have a better footage when absolute quantification was performed. In this context, two commercially available wheat samples, whole meal and extracted wheat flour type 405 were analyzed. The analytical method used for the ATI analysis of wheat samples was the one developed in our previous work [20]. Compared to the average ATI contents in the two wheat samples, the results revealed that the relative amounts of ATIs were 2–3 times lower in sorghum samples SOR 1099, B-Sc, SOR 1236, SOR 958, SOR 20 and SOR 557 and up to 60 times lower in nine of the analyzed sorghum cultivars (SOR 777, SOR 618, R-Sc, SOR 654, SOR 759, SOR 226, SOR 1055, SOR 844 and SOR 555); while considering the corresponding averages (Figure 4a). The relatively low levels of ATIs in sorghum might suggest that sorghum could be a good alternative. However, as there was not enough absolute quantification data for comparison available here and also in the literature, it would be interesting to investigate this aspect further.

## 3. Materials and Methods

### 3.1. Materials

#### 3.1.1. Food Material

Fifteen sorghum cultivars from different regions were analyzed, including five samples from Africa (accession number: SOR 557, SOR 1055, SOR 555, B. Sc and R. Sc); four from Asia (accession number: SOR 226, SOR 618, SOR 759 and SOR 844); three from America (accession number: SOR 777, SOR 1099 and SOR 1236) and three Europe (accession number: SOR 654, SOR 20 and SOR 958). In total, fourteen sorghum samples were cultivars from *Sorghum bicolor* (L.) Moench, and one from *Sorghum halepense* (L.) Pers. Samples B.Sc and R.Sc were obtained from the Institute of Research and Agronomic Development, IRAD (Maroua, Cameroon) while the other ones were provided by the Leibniz Institute for Plant Genetics and Crop Plant Research (Gatersleben, Germany). Table 1 summarizes information on sorghum samples. The sampling was carried out in order to obtain a global representation of the different sorghum produced for human consumption around the world and to compare them.

Further, two commercially available wheat samples-whole meal (Biokorn GmbH and Co. KG, Aalen, Germany) and extracted wheat flour type 405 (Kunstmühle Reisgang, Pfaffenhofen, Germany) were used to compare the relative content of ATIs in sorghum samples to those of wheat flour.

#### 3.1.2. Standards and Chemicals

A short peptide with four amino acids (GWGG) and a ratio *m/z* of 376.2 provided by Bachem AG (Bubendorf, Switzerland) was used as an internal standard (IS) for the MS analysis. Proteomics grade trypsin and pepsin (Sigma Aldrich, Steinheim, Germany) were used to perform the protein digestion. Whey protein containing ca. 93.4% of β-lactoglobulin (Davisco Foods International, Le Sueur, MN, USA) was used to evaluate the efficiency of the trypsin digestion and check the matrix effect. All the other chemicals were of analytical grade.

### 3.2. Methods

**Figure 5:**
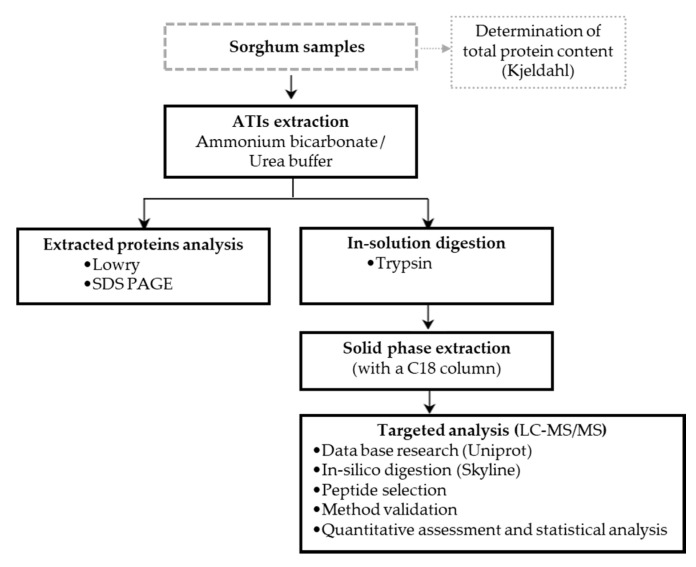
Workflow showing the different processing steps and the analytical methods.

Figure 5 presents the workflow of the respective processing steps. Sorghum samples were first analyzed in order to determine their protein content according to Kjeldahl. Extraction was then performed using the flour samples following by the trypsin digestion. The solid phase extraction (SPE) was used to clean the samples after which LC–MS/MS analyses were carried out.

#### 3.2.1. Protein Extraction and In-Solution Digestion

The protein extraction and the in-solution digestion steps were based on the method established and optimized in our previous work [20] using ammonium bicarbonate (100 mM) buffer containing urea (4 M). Figure S7 (Supplementary Materials) provides the different extraction steps. Briefly, 100 mg flour was weighed twice into a 2 mL reaction tubes and 1 mL of extraction buffer was added. The mixture was shaken at 95 rpm for one hour and then centrifuged at 7000× *g*, 4 °C for ten minutes. 0.4 mL of supernatants were then collected and transferred to a 1.5 mL reaction tube to perform the in-solution digestion. Of β-lactoglobulin (0.5 mg/mL) 10 µL was added in the sample and in 0.4 mL of extraction buffer as control in order to check the efficiency of the in-solution digestion. Initially four different reducing (tris (2 carboxyethyl) phosphine-TCEP, dithiothreitol-DTT, tris(hydroxypropyl)phosphine-THPP and β-mercaptoethanol-BME) and three alkylating agents (iodoacetamide-IAA, N-ethylmaleimide-NEM and 4-vinylpyridine-4VP) were tested to optimize the digestion conditions and the recoveries for the peptides were compared. Concentrations and conditions used for these steps were in the order of those given for TCEP and IAA below while using distilled water as solvent. Based on these preliminary experiments, the following final procedure was adapted for all samples. The reduction was thereafter consistently done by adding 10 µL of 0.25 M TCEP followed by a twenty-minute incubation at 50 °C was performed. The samples were then mixed with 10 µL of 0.25 M of IAA and incubated in the dark for 20 min at 50 °C. Then 135 µL of digestion buffer (100 mM ammonium bicarbonate) and 20 µL of 4 mg/mL trypsin solution were given to the samples. The digestion was performed overnight at 37 °C under the shaking conditions (300 rpm) using a thermoshaker (BioShake iQ, Analytik Jena™, Jena, Germany). Finally, 15 µL of a 40% formic acid (*v*/*v*) was added to denature the enzyme and stop the digestion.

#### 3.2.2. Solid Phase Extraction

Solid phase extraction (SPE) was used to achieve purification and enrichment of analytical samples after the in-solution digestion. The column constituted of 300 mg of C18 material (Chromabondfi sorbent C18 ec, Macherey-Nagel GmbH and CO. KG, Düren, Germany) was first conditioned using 6 mL of solvent A (acetonitrile/distilled water/formic acid, 50/50/0.1%, *v*/*v*) and then conditioned with 6 mL of bidistilled water. The aim of these 2 steps was to build a balance between liquid and solid phases and to remove possible impurities. 2 × 600 µL of the digested samples were then applied to the column and run at a low flow rate to allow the peptides to bind to the column properly. Afterwards, the interfering components were removed by washing the column with 6 mL bidistilled water. The analytes were finally eluted from the column using 1 mL of solvent B (200 mL acetonitrile and 200 µL formic acid, 0.1%) and the volume was filled to 5 mL with bidistilled water by giving water thru the column. Samples were then stored at −20 °C and were mixed with the internal standard just prior to MS analysis.

#### 3.2.3. Protein Determination

Samples collected after the extraction steps were analyzed to determine the concentration of extracted protein used for the in-solution digestion. For this purpose, Lowry method [33] was applied with BSA as standard. The data obtained were subsequently used to express the final results from the LC–MS/MS analysis as a function of the initial sample mass and of the amount of extracted protein.

#### 3.2.4. Sodium Dodecyl Sulfate Polyacrylamide Gel Electrophoresis (SDS PAGE)

SDS PAGE was performed according to Laemmli [51]. Initially, 40 μL of the proteins extract were incubated with 8 μL sample buffer (0.106 M Tris-HCl buffer at pH 8.5, containing 0.51 mM of EDTA, 2% of lithium dodecyl sulfate, 10% of glycerol, 0.22 mM of Coomassie Blue G 250 and 0.175 mM of Phenol Red, Thermo Fischer Scientific, Waltham, E.U.A.) at 90 °C for 5 min. After that, 20 μL of the solution and 5 μL of the standard (Page Ruler^TM^ Prestained Protein Ladder (10–250 kDa), Thermo Fischer Scientific, Waltham, MA, USA) were separated by SDS-PAGE (NuPAGE^TM^ 12% Bis-Tris Gel, Thermo Fischer Scientific, Waltham, MA, USA) by loading the solution, followed by staining with Coomassie Brilliant blue R250 (in 10% acetic acid) and destaining with 10% acetic acid. After scanning the gels, the band intensity and the relative molecular weight of proteins were estimated using Image Lab software (version 6.0.1.34, Bio-Rad Laboratories, Hercules, CA, USA).

#### 3.2.5. LC–MS/MS Method Development and Validation

The development of a suitable LC–MS/MS method was essential for the detection of ATIs from sorghum. Therefore, settings for mass spectrometric analysis had to be defined and optimized beforehand. As no data about the MS analysis of sorghum ATIs were available, the method development was done by using the empirical approach consisting of analyzing step by step the procedures consisting of four different steps, including the identification and selection of proteins of interest, generation and selection of the corresponding peptides with their corresponding MS parameters, the determination of the retention time and the optimization of the collision energy.

##### Method Development

Information about protein sequences of sorghum ATIs was extracted from the online UniProt Knowledgebase (UniProtKB; https://www.uniprot.org). As of 15.11.2019, the database contained two different entries for sorghum ATIs: proteins P81367 and P81368; both reviewed. Table 3 gives the main information about these two sorghum ATIs. Their primary sequences were downloaded from the UniProt database as FASTA format and imported into Skyline software (MacCoss Lab Software, version 20.2, University of Washington, Seattle, WA, USA; https://skyline.gs.washington.edu) [34]. This approach has been detailed in our former work [20].

The sequences of both proteins were compared by aligning them with Jalview version 2.8, an open source bioinformatics software [52]. It indicates that they are 33.6% identical, with 41 amino acid residues in identical position. The literature was checked and no data was found about comparable analysis.

Subsequently, the imported protein sequences were theoretically digested with Skyline. The editing peptide settings was done by selecting trypsin as enzyme, zero missed cleavage and carbamidomethyl of cysteine residue as structural modification. Likewise, precursor charges 2, 3; ion charge 1; and ion types y and b were set. Peptides were further filtered to include only those with a length between 5 and 30 amino acids. For each peptide, several fragment transitions were initially tested and 3–4 transitions with the highest intensities were selected. The program generated a summary of the peptides produced by the in silico digestion. Optimization of the HPLC gradient and the MS instrument were performed to improve the separation of peptides and the collision energies. At the development stage, many peptides were analyzed and finally one biomarker was selected per protein for the relative quantification-peptides unique to each sorghum ATI molecule. In addition, we selected two more peptides per protein to serve as qualitative control during the analysis. The specificity of the generated peptides was determined with the BLAST function (Basic Local Alignment Search Tool) [53]. At least three transitions per peptide were selected during the development of the method, with the highest possible signal intensity. Skyline was also used for subsequent optimization of the peptide and transition selection. A detailed summary of the investigated sorghum ATIs and their peptides can be found in Table S1 (Supplementary Materials).

Prior to the analysis, the digested samples were mixed with the internal standard (GWGG) to get a final concentration of 0.01 µg/mL). The analysis was performed with an LC Agilent Infinity 1260 System (consisting of a binary pump, a multi-column thermostat, a VL vial sampler) coupled with a mass spectrometer Agilent G6470A Series Triple Quad HPLC/MS (Agilent Technologies Sales and Services GmbH and Co.KG, Waldbronn, Germany). Kinetex C8 column (150 mm × 4.6 mm; 2.6 µm; Phenomenex, Torrance, CA, USA) was used and the separation was performed at a flowrate of 0.5 mL/min for total time of 32 min (including 28 min of separation and 4 min of postrun) and under the gradient conditions with 0.1% formic acid and acetonitrile as solvent A and B, respectively. The elution program was as follow: 0.01–2.0 min, 5% solvent B; 18 min, 50% solvent B; 19 min, 95% solvent B; 22 min, 95% solvent B; 23 min, 5% solvent B and 28 min, 5% solvent B. The tandem mass spectrometry conditions (MS/MS) parameters were collision gas: nitrogen; pressure: 3.85E-5 Torr; detection: multiple reaction monitoring (MRM); fragmentor voltage: 130 V; cell accelerator voltage: 5 kV and dwell time: 20 ms. Collision energy (CE, eV) was individually optimized for each analyzed peptide. The electrospray ionization (ESI) was performed in a positive ion-mode at a desolvation temperature of 275 °C using nitrogen as desolvation gas (flow-rate: 11.0 L/min); a capillary current of 5425 nA and the nebulizer pressure of 35.0 psi.

In order to monitor the digestion process and to assess the effect of the matrix, solution of β-lactoglobulin was incorporated into the sorghum samples just before the digestion. A blank was also constituted using distilled water instead of sample extracts. All assays were performed in triplicate. A biomarker peptide was selected for each protein to perform the relative quantification using their total peak areas. The averages of the values obtained were normalized using the internal standard peptide. The amount of β-lactoglobulin in the blank was finally compared to those in the samples.

##### Method Validation

The developed LC–MS/MS method was validated before being used to perform the relative quantification of ATIs in different sorghum samples. For this purpose, 5 of the 15 sorghum samples were first used for to validate the developed method. Different parameters were analyzed, including the effect of the matrixes on the β-lactoglobulin, the recovery of the internal standard in sample matrixes, the repeatability/reproducibility (intraday/interday analysis), linearity and detection and quantification limits.

The matrix effect was assessed by checking and comparing the IS response in water with the IS responses in different sample matrices and the results were expressed as % recovery. Repeatability was achieved by repetitive analysis of the IS and samples within the same day (intraday) while reproducibility was achieved between several days/weeks (interday) by repeating the entire process from extraction to LC–MS/MS analysis. Linearity was evaluated at several protein concentration ranges by injecting different amounts of IS and samples as triplicates into the LC–MS system. The IS/protein peak areas were then represented as a function of the corresponding protein concentrations and the coefficient of determination (*R*^2^) calculated from the regression curve was used to estimate linearity.

The limit of detection (LOD) is the lowest amount of content that can be measured with statistical confidence while the limit of quantification (LOQ) is the lowest amount of analyte that can be quantitatively determined with precision and accuracy while considering the effect of the matrix [54]. The linear regression method (y=a + bx) according to the International Conference on Harmonization (ICH) was used to determine the limit of detection $\left(LOD=3S_{a}/b\right)$ and the limit of quantification $\left(LOQ=10S_{a}/b\right)$. With $S_{a}$ the standard deviation of the response and b, the slope of the regression curve.

#### 3.2.6. Analysis of Wheat Samples

As mentioned previously, we performed the analysis of two commercial wheat samples in order to compare their ATIs content with those of the sorghum samples. The complete procedure of the wheat ATIs extraction using ammonium bicarbonate/urea as buffer and their relative quantification by LC–MS/MS was performed according to our previous work [20]. In total, fourteen wheat ATIs (P01083, P17314, P16850, P01084/85, P15851, P16159, P81496/Q43723/Q43691, P93602, P83207, Q4U199 and Q41540) were identified and analyzed in both wheat samples. Table S2 (Supplementary Materials) summarizes biomarkers used as quantifier peptides and their optimized analytical conditions.

#### 3.2.7. Quantitative Assessment and Statistical Analysis

The results of the LC–MS/MS were expressed as peak area (PA) and PA/mg flour. All the experiments were performed in three independent replicates and data were reported as mean plus the standard deviation. Statistical analyses were carried out using GraphPad Prism 6 (version. 6.01, GraphPad Inc., San Diego, CA, USA) and data were considered statistically significant when the averages of the compared results differed at a 5% of the significance level (when not specified). 

## 4. Conclusions

The objective of this work was to contribute to the characterization and relative quantification of sorghum ATIs by targeted LC–MS/MS. Fifteen sorghum cultivars from different regions were analyzed and two wheat samples were used to compare their ATI contents. The Ambi/urea extract showed different proteins content using the Lowry method. Protein composition was assessed by SDS PAGE and samples presented different profiles. The sample preparation and LC–MS/MS method were developed and optimized using β-lactoglobulin and internal standard to monitoring the digestion and MS analysis. ELAAVPSR (*m/z*: 907.4613) and TYMVR (*m/z*: 335.1731) were identified and retained as biomarkers to carry out the relative quantification of ATIs P81367 and P81368, respectively. Combination of TCEP and IAA yielded the best intensities in term of quantifier peptides and cysteine-containing peptides. It is appeared that sample matrix affected significantly β-lactoglobulin during the tryptic digestion while the internal standard recovery was found to be in the ranges of 92.2% and 107.6%. The linearity and the repeatability/reproducibility of the method were demonstrated. Sorghum samples showed different trends in term of relative ATIs content and cultivar SOR 777 from Mexico, representing the unique sorghum cultivar from *S. halepense* exhibited the lowest amount of ATIs while comparing it to all the other cultivars from *S. bicolor* species. This raised in addition to the questions of the cultivation patterns in the different growing regions, also the issue of the species of the sorghum cultivars, which could potentially play an important role while considering the ATI contents. Comparing the data to the average of ATIs in parallel analyzed wheat samples, it emerged that six sorghum cultivars presented 2–3 times lower ATI than wheat. This was up to 60 times lower in nine sorghum cultivars. Thereafter, the relatively low levels of ATIs in sorghum might suggest that sorghum can be a good alternative. This is a preliminary assessment and should be reinforced by the absolute quantification of sorghum ATIs in comparison to wheat. Unfortunately the corresponding immune-toxicological response has not yet been documented for sorghum cultivars and still needs to be addressed. It would be interesting to investigate these aspects further including the targeting of the absolute quantification of sorghum ATIs.

## Figures and Tables

**Table 1:** Selected sorghum cultivars used for α-amylase/trypsin inhibitor (ATI) analysis and their origins.

No.	Accession Number	Accession Name	Scientific Name	Race	Country of Origin	Region	Total Proteins * (g/100 g)	Extracted Proteins ** (mg/mL)
1	B.Sc	S35	*Sorghum bicolor* (L.) Moench	Madjeru	Cameroon	Africa	7.9	2.137 ± 0.139 ^a^
2	R.Sc	Damougari	*Sorghum bicolor* (L.) Moench	Safari	Cameroon	Africa	8.5	2.170 ± 0.132 ^a,c^
3	SOR 557	n.a.	*Sorghum bicolor* (L.) Moench	Durra	Egypt	Africa	13.5	2.684 ± 0.145 ^b^
4	SOR 1055	HAMRA	*Sorghum bicolor* (L.) Moench	Kafir-Durra	Yemen	Africa	15.4	2.553 ± 0.013 ^b^
5	SOR 555	H × 60	*Sorghum bicolor* (L.) Moench	Kafir-Bicolor	Tansania	Africa	13.6	2.506 ± 0.066 ^a,b^
6	SOR 777	n.a.	*Sorghum halepense* (L.) Pers.	n.a.	Columbia	America	14.4	4.046 ± 0.106 ^c^
7	SOR 1099	BATAN73 620	*Sorghum bicolor* (L.) Moench	Guinea	Mexico	America	17.0	2.049 ± 0.026 ^a^
8	SOR 1236	WRAY	*Sorghum bicolor* (L.) Moench	Caudatum	USA	America	12.2	2.506 ± 0.198 ^a,b^
9	SOR 226	Jan-Da-Li	*Sorghum bicolor* (L.) Moench	Durra	China	Asia	12.8	3.719 ± 0.238 ^c^
10	SOR 618	n.a.	*Sorghum bicolor* (L.) Moench	Bicolor	n.a.	Asia	12.2	4.140 ± 0.066 ^c^
11	SOR 759	n.a.	*Sorghum bicolor* (L.) Moench	Bicolor	Korea, PRK	Asia	11.3	2.343 ± 0.059 ^a,b^
12	SOR 844	n.a.	*Sorghum bicolor* (L.) Moench	Guinea	Iraq	Asia	12.1	0.588 ± 0.047 ^d^
13	SOR 654	Blackhull	*Sorghum bicolor* (L.) Moench	Durra-Bicolor	United Kingdom	Europe	12.1	3.383 ± 0.238 ^c^
14	SOR 20	n.a.	*Sorghum bicolor* (L.) Moench	Durra	Denmark	Europe	14.7	2.474 ± 0.125 ^a,b^
15	SOR 958	Tech 8	*Sorghum bicolor* (L.) Moench	Bicolor	Germany	Europe	14.0	3.048 ± 0.026 ^b,c^

n.a. not available. Lowry data are expressed as means ± standard deviation, *n* = 3. Different letters indicate significantly different values (*p* < 0.05). * total protein content of sorghum flours according to Kjeldahl method (N × 6.25); ** amount of proteins from the ammonium bicarbonate/urea extraction according to the Lowry method.

**Table 2:** Summary of method validation results. For ATIs P81367 and P81368, the results from cultivar SOR 958 are presented.

Validation Criterion	IS-H_2_O (GWGG)	β-Lg (GLDIQK)	P81367 (ELAAVPSR)	P81368 (TYMVR)
IS recovery (%)	100.0 ± 0.5	104.1 ± 0.6	99.4 ± 0.9	99.4 ± 0.9
Repeatability (% RSD)	1.98	0.61	1.50	0.41
Reproducibility (% RSD)	1.42	1.03	2.32	1.17
Linearity ($R^{2}$)	0.9973	0.9992	0.9995	0.9968
LoD (ng protein)	0.075 *	0.001	0.048	0.346
LoQ (ng protein)	0.250 *	0.004	0.161	1.155

IS = internal standard and β-Lg = β-lactoglobulin. RSD = relative standard deviation, LoD = limit of detection, LoQ = limit of quantification. The asterisk (*) indicates that LoD and LoQ values of the IS are given in pg of the peptide.

**Table 3:** List of sorghum α-amylase/trypsin inhibitor available from the online database UniProtKB, as of 15 November 2019. Both proteins are reviewed. MW is the molecular weight, pI the isoelectric point and AA is the number of amino acid residues.

Entry	Protein Name	Short Name	MW (Da)	pI	Length (AA)
P81367	Alpha-amylase inhibitor 4	IAA4_SORBI	12,449.45	8.96	118
P81368	Alpha-amylase inhibitor 5	IAA5_SORBI	12,776.11	8.55	116

## Supplementary Materials

The following are available online, Figure S1: SDS PAGE of some sorghum cultivars, Figure S2: Comparison of different reducing agents with all selected peptides from ATIs P81367, P81368 and β-Lg (β-lactoglobulin), Figure S3: Comparison of different alkylating agents with all selected peptides from ATIs P81367, P81368 and β-Lg (β-lactoglobulin), Figure S4: Recovery of the internal standard in blank and in the samples, Figure S5: Intraday (repeatability) and interday (reproducibility) analysis of ATIs (a) P81367 and (b) P81368 using samples JDL, Tech 8, Wray, HX-60 and B-SC, Figure S6: Linearity of the measurements of the (a) internal standard, (b) β-bactoglobulin and quantifier peptides of proteins (c) P81367 and (d) P81368, Figure S7: Flowsheet presenting the different steps of the sample preparation prior their LC–MS/MS analysis, Figure S8: Sequence alignment of the two P81367 and P81368 sorghum ATIs, Table S1: The optimized conditions of the LC–MS/MS method for the analysis and relative quantification of ATIs in sorghum samples, Table S2 The optimized conditions for the multiple reaction monitoring (MRM) for the analysis of wheat samples.
