# Supplementary material for: Relative Abundance of Alpha-Amylase/Trypsin Inhibitors in Selected Sorghum Cultivars
Source: Molecules. 2020 Dec 17;25(24):5982. doi: 10.3390/molecules25245982 (PMC7774315; doi:10.3390/molecules25245982)
Supplement: Supplementary file 1 [file molecules-25-05982-s001.pdf]

Supplementary Material

Relative abundance of alpha-amylase/trypsin inhibitors in selected sorghum cultivars

Sorel Tchewonpi Sagu[1]. Eva Landgräber[1]. Michal Rackiewicz[1]. Gerd Huschek[2] and Harshadrai M. Rawel[1.*]

1. Institute of Nutritional Science, University of Potsdam, Arthur-Scheunert-Allee 114-116. 14558 Nuthetal, Potsdam. Germany; sorelsagu@uni-potsdam.de (S.T.S.); e.landgraeber@live.de (E.L); rackiewicz@uni-potsdam.de (M.R.)
2. IGV-Institut für Getreideverarbeitung GmbH, Arthur-Scheunert-Allee 40/41. D-14558. Nuthetal OT Bergholz-Rehbrücke, Germany; gerd.huschek@igv-gmbh.de (G.H.)

* Corresponding author.

Tel: +49-33200-88-5525/5578 (H.R.)

E-mail: rawel@uni-potsdam.de

Content

Figure S1 SDS PAGE of some sorghum cultivars. The analysis was performed under denaturation conditions using 12% Bis-Tris Gel.

Figure S2 Comparison of different reducing agents with all selected peptides from ATIs P81367, P81368 and β-Lg (β-lactoglobulin). Iodoacetamide was used as alkylating agent. Reducing agents: TCEP - Tris(2 carboxyethyl)phosphine; DTT - Dithiothréitol; THPP - Tris(hydroxypropyl)phosphine; BME - beta-mercaptoethanol

Figure S3 Comparison of different alkylating agents with all selected peptides from ATIs P81367, P81368 and β-Lg (β-lactoglobulin). Tris(2 carboxyethyl)phosphine (TCEP) was used as reducing agent. Alkylating Agents: IAA – Iodoacetamide; 4-VP – 4-vinylpyridine

Figure S4. Recovery of the internal standard in blank and in the samples. Experiments 1, 2, 3 and 4 were performed on different days and in triplicate.

Figure S5. **I**ntraday (Repeatability) and interday (reproducibility) analysis of ATIs (a) P81367 and (b) P81368, using samples JDL, Tech 8, Wray, HX-60 and B-SC

Figure S6. Linearity of the measurements of the (a) internal standard, (b) β-bactoglobulin and quantifier peptides of proteins (c) P81367 and (d) P81368.

Figure S7. Flowsheet presenting the different steps of the sample preparation prior their HPLC-MS/MS analysis

Figure S8. Sequence alignment of the two P81367 and P81368 sorghum ATIs

Table S1 The optimized conditions of the HPLC-MS/MS method for the analysis and relative quantification of ATIs in sorghum samples.

Table S2 The optimized conditions for the multiple reaction monitoring (MRM) for the analysis of wheat samples. Q1 = Precursor mass; Q3 = Transition mass; CE = Collision energy

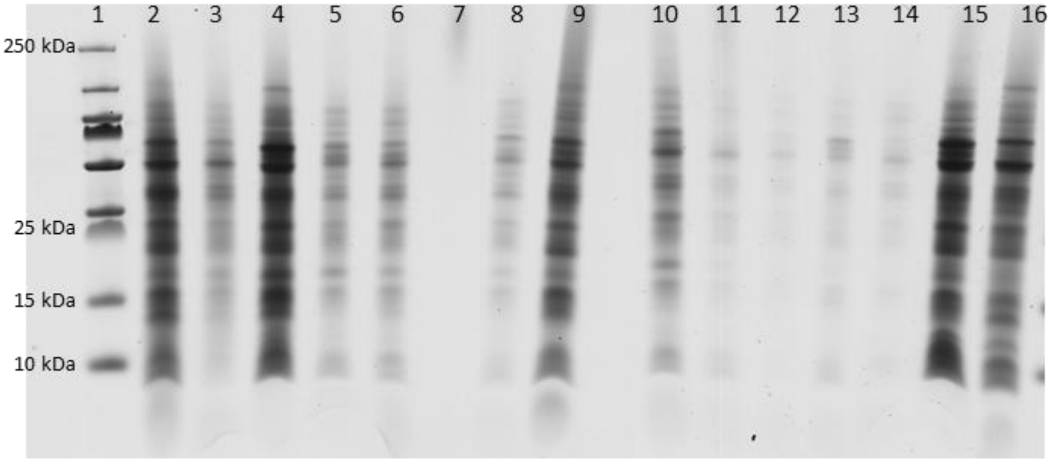

Figure S1 SDS PAGE of some sorghum cultivars. The analysis was performed under denaturation conditions using 12% Bis-Tris Gel. 1 is protein ladder and 2 to 16 are samples B-Sc,R-Sc, SOR 557, SOR 1055, SOR 555, SOR 777, SOR 1099, SOR 1236, SOR 226, SOR 618, SOR 759, SOR 844, SOR 654, SOR 20 and SOR 958, respectively.

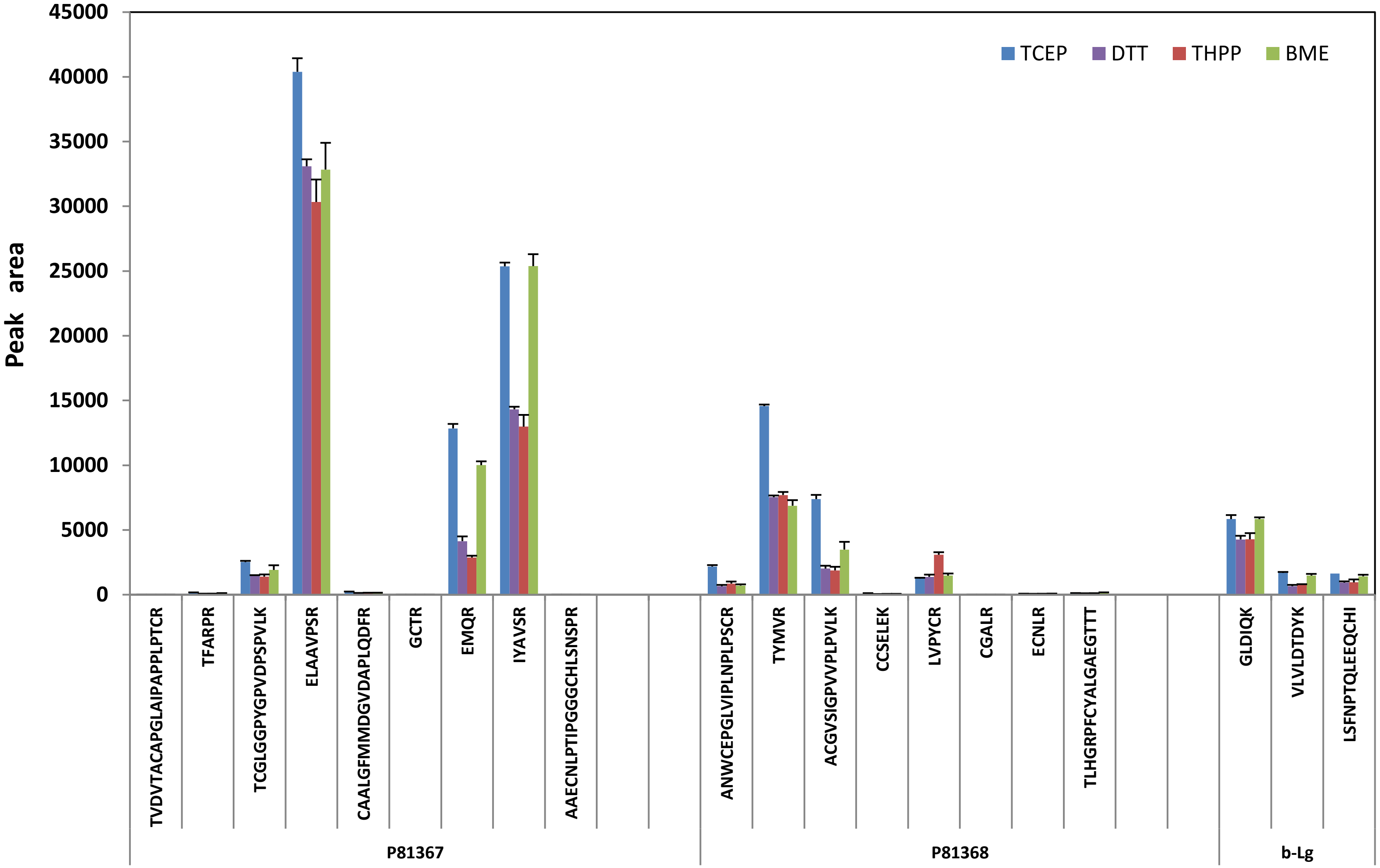

Figure S2 Comparison of different reducing agents with all selected peptides from ATIs P81367, P81368 and β-Lg (β-lactoglobulin). Iodoacetamide was used as alkylating agent. Reducing agents: TCEP - Tris(2 carboxyethyl)phosphine; DTT - Dithiothréitol; THPP - Tris(hydroxypropyl)phosphine; BME - beta-mercaptoethanol

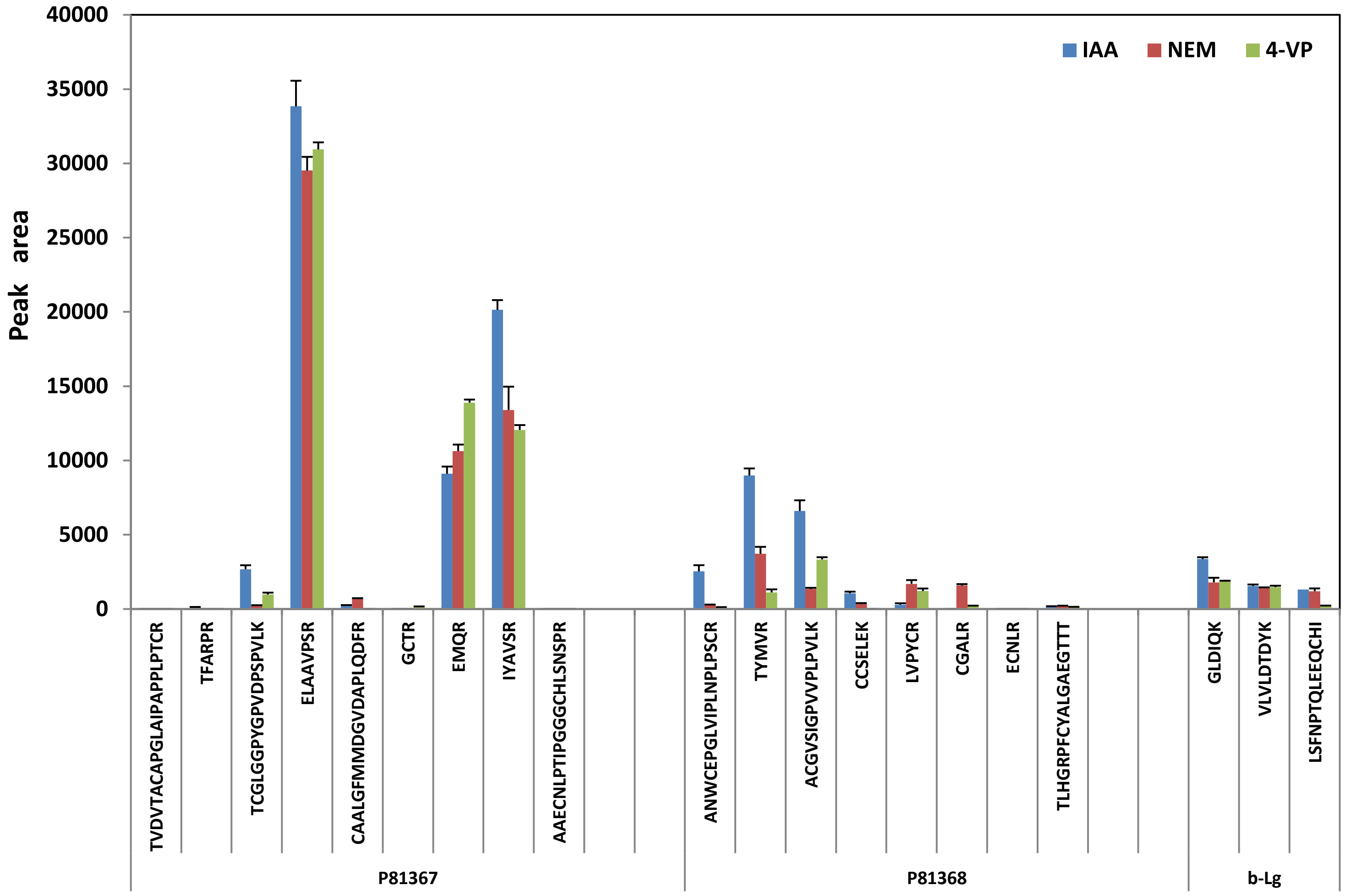

Figure S3 Comparison of different alkylating agents with all selected peptides from ATIs P81367, P81368 and β-Lg (β-lactoglobulin). Tris(2 carboxyethyl)phosphine (TCEP) was used as reducing agent. Alkylating Agents: IAA – Iodoacetamide; 4-VP – 4-vinylpyridine

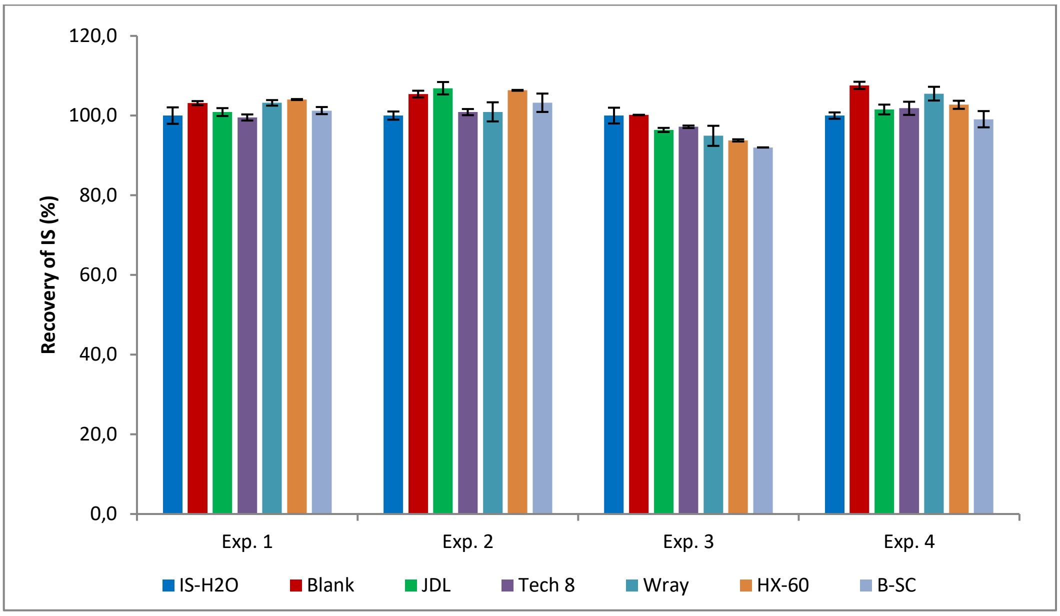

Figure S4. Recovery of the internal standard in blank and in the samples. Experiments 1, 2, 3 and 4 were performed in different days and in triplicate.

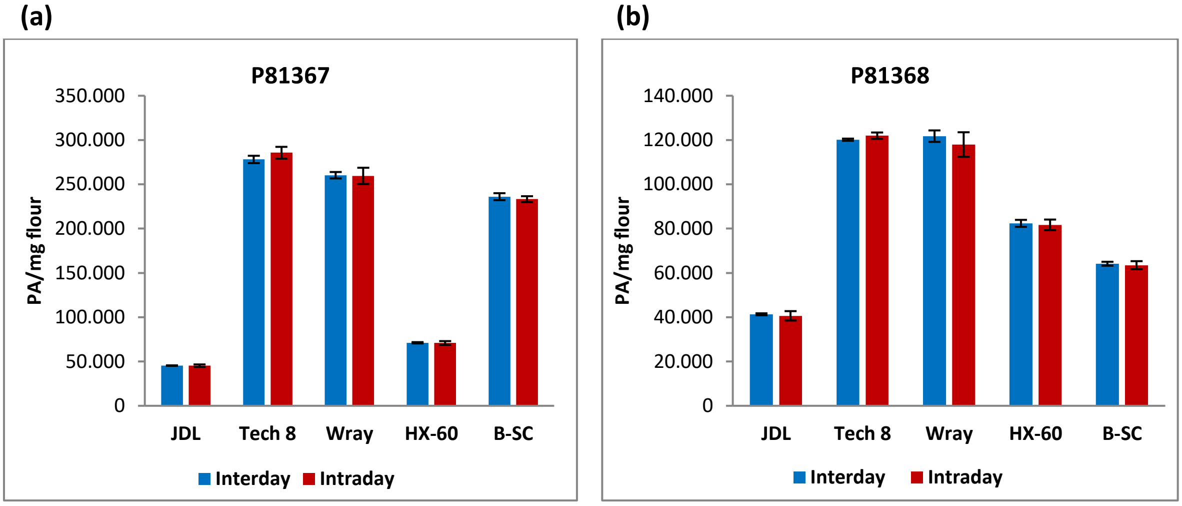

Figure S5. **I**ntraday (Repeatability) and interday (reproducibility) analysis of ATIs (a) P81367 and (b) P81368 using samples JDL, Tech 8, Wray, HX-60 and B-SC

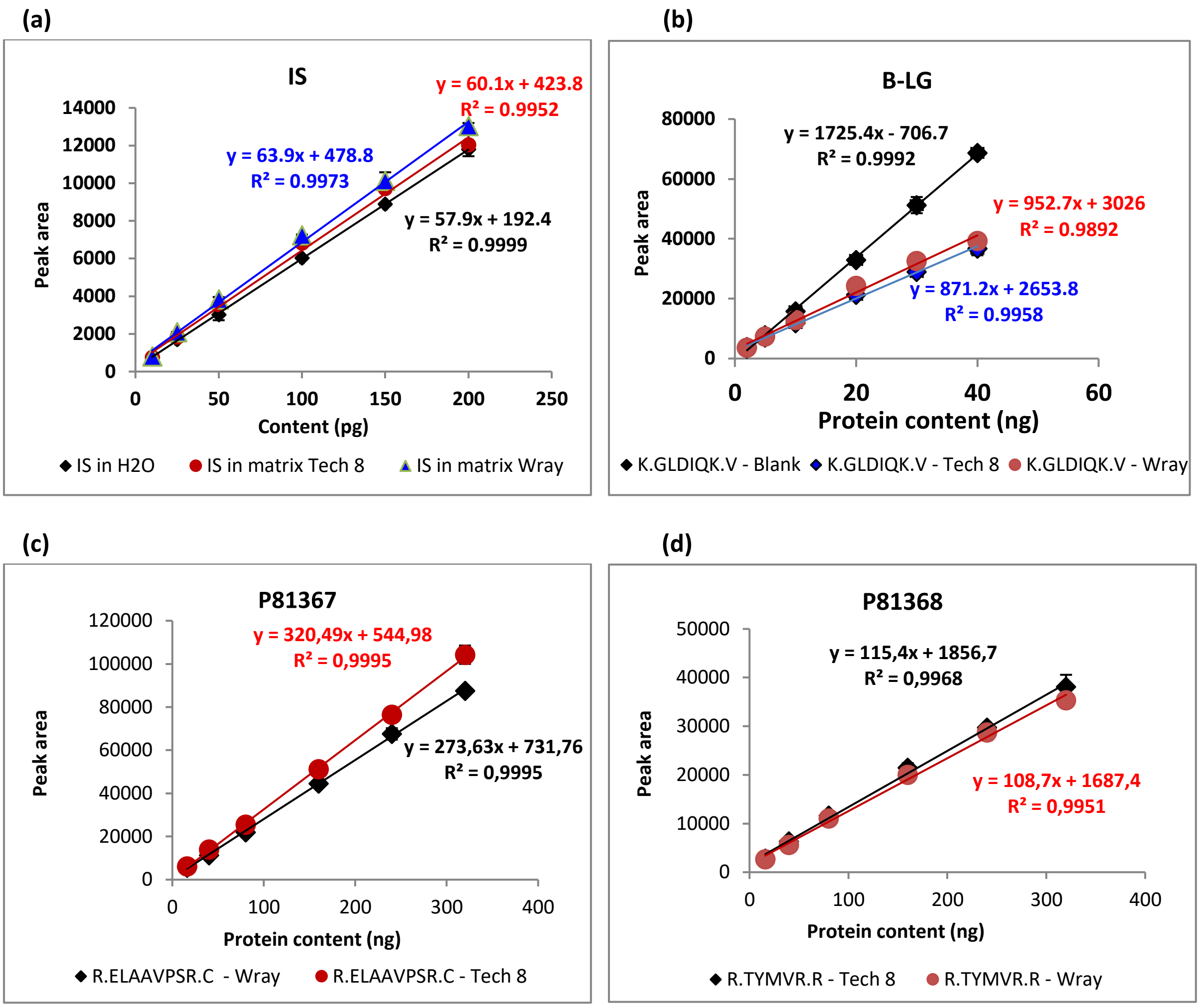

Figure S6. Linearity of the measurements of the (a) internal standard, (b) β-bactoglobulin and quantifier peptides of proteins (c) P81367 and (d) P81368.

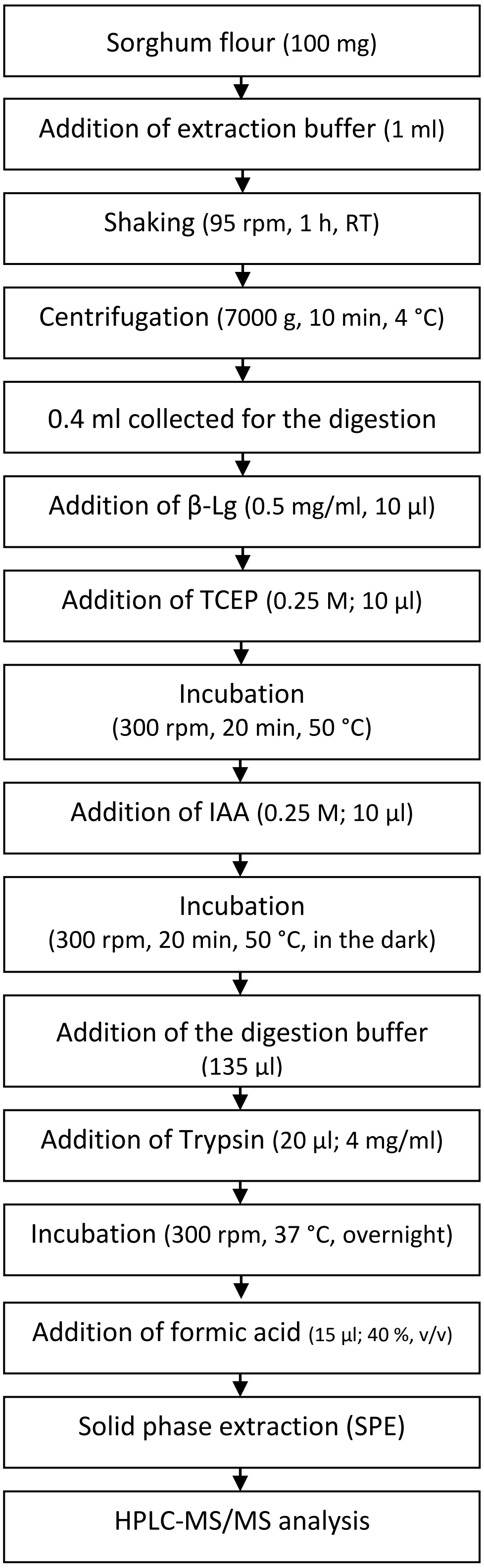

Figure S7. Flowsheet presenting the different steps of the sample preparation prior their HPLC-MS/MS analysis

```
SP|P81367|IAA4_SORBI/1-118     1 T V D V T A C A P G L A I P A P P L P T C R T F A R P R T 29
SP|P81368|IAA5_SORBI/1-116     1 - - - A N W C E P G L V I P L N P L P S C R T Y M V R R A 26

SP|P81367|IAA4_SORBI/1-118    30 C G L G G P Y G P V D P S P V L K Q R C C R E L A A V P S 58
SP|P81368|IAA5_SORBI/1-116    27 C G V - - S I G P V V P L P V L K E R C C S E L E K L V P 53

SP|P81367|IAA4_SORBI/1-118    59 R C R C A A L G F M M D G V D A P L Q D F R G C T R E M Q 87
SP|P81368|IAA5_SORBI/1-116    54 Y C R C G A L R T A L D S M M T G Y E M R P T C S W G G L 82

SP|P81367|IAA4_SORBI/1-118    88 R I Y A V S R L T R A A E C N L P T I P G G G C H L S N S 116
SP|P81368|IAA5_SORBI/1-116    83 L T F - A P T I V C Y R E C N L R T L H G R P F C Y A L G 110

SP|P81367|IAA4_SORBI/1-118   117 P R - - - -                                               118
SP|P81368|IAA5_SORBI/1-116   111 A E G T T T                                               116
```

Figure S8. Sequence alignment of the two P81367 and P81368 sorghum ATIs

Table S1 The optimized conditions of the HPLC-MS/MS method for the analysis and relative quantification of ATIs in sorghum samples.

Protein	Sequence	Q1-Mass	Fragment	Q3-Mass	CE [eV]	Retention time (min)
P81367	R.ELAAVPSR.C	421.7^{++}	A [y6]	600.3^{+}	14.1	8.6
			A [y5]	529.3^{+}	17.1	
			V [y4]	458.3^{+}	11.1	
			P [y3]	359.2^{+}	11.1	
	TVDVTACAPGLAIPAPPLPTCR.T	n.d.		n.d.		n.d.
	R.TFARPR.T	374.2^{++}	A [y4]	499.3^{+}	9.6	8.3
			F [b2]	249.1^{+}	12.6	
			A [b3]	320.2^{+}	12.6	
	R.TCGLGGPYGPVDPSPVLK.Q	907.5^{++}	P [y9]	951.6^{+}	29.1	11.7
			P [y6]	640.4^{+}	35.1	
			P [y4]	456.3^{+}	35.1	
			G [b6]	546.2^{+}	26.1	
	R.CAALGFMMDGVDAPLQDFR.G	1057.4^{++}	V [y9]	1060.5^{+}	33.8	14.5
			D [y8]	961.4^{+}	33.8	
			P [y6]	775.4^{+}	33.8	
			V [b11]	1153.1^{+}	33.8	
	R.EMQR.I	282.1^{++}	Q [y2]	303.2^{+}	9.7	9.6
			R [y1]	175.1^{+}	9.7	
			M [b2]	261.1^{+}	9.7	
	R.G**C**TR.E	n.d.		n.d.		n.d.
	R.AAECNLPTIPGGGCHLSNSPR.	n.d.		n.d.		n.d.
	R.IYAVSR.L	354.7^{++}	Y [y5]	595.3^{+}	12.0	8.2
			A [y4]	432.3^{+}	9.0	
			V [y3]	361.2^{+}	12.0	
			A [b3]	348.2^{+}	3.0	
P81368	R.TYMVR.R	335.2^{++}	Y [y4]	568.3^{+}	14.4	8.2
			M [y3]	405.2^{+}	8.4	
			Y [b2]	265.1^{+}	6.4	
	-.ANW**C**EPGLVIPLNPLPS**C**R.T	1097.0^{++}	I [y10]	1166.6^{+}	35.0	13.8
			P [y9]	1053.6^{+}	35.0	
			P [y6]	729.3^{+}	35.0	
			P [y4]	519.2^{+}	35.0	
			E [b5]	661.2^{+}	35.0	
	R.**CC**SELEK.L	463.1^{++}	E [y4]	518.3^{+}	15.4	7.0
			L [y3]	389.2^{+}	15.4	
			S [b3]	408.1^{+}	15.4	
			E [b4]	537.1^{+}	15.4	
	R.A**C**GVSIGPVVPLPVLK.E	803.4^{++}	P [y9]	961.6^{+}	25.9	13.7
			V [y7]	765.5^{+}	25.9	
			P [y6]	666.4^{+}	25.9	
			P [y4]	456.3^{+}	25.9	
	R.**C**GALR.T	n.d.		n.d.		n.d.
	K.LVPY**C**R.C	404.2^{++}	V [y5]	694.3^{+}	13.5	8.5
			P [y4]	595.3^{+}	16.5	
			Y [y3]	498.2^{+}	4.5	
	R.E**C**NLR.T	346.2^{++}	C [y4]	562.3^{+}	14.7	6.3
			N [y3]	402.3^{+}	11.7	
			N [b3]	404.1^{+}	14.7	

	R.TLHGRPF**C**YALGAEGTTT.-	651.3^{++}	G [y7]	636.3^{+}	21.6	13.9
			A [y6]	579.3^{+}	24.6	
			F [y12]	645.8^{+}	9.6	
			C [y11]	572.3^{+}	21.6	
**b-Lg**	K.GLDIQK.V	337.1^{++}	D [y4]	503.2^{+}	8.5	8.5
			I [y3]	388.2^{+}	11.5	
			Q [y2]	275.1^{+}	14.5	
	K.VLVLDTDYK.K	533.2^{++}	L [y6]	754.3^{+}	14.5	11.1
			D [y5]	641.2^{+}	14.5	
			T [y4]	526.2^{+}	23.5	
	R.LSFNPTQLEEQCHI.	858.4^{++}	P [y10]	1254.5^{+}	30.0	12.4
			Q [y8]	1056.4^{+}	30.0	
			L [y7]	928.4^{+}	30.0	
			E [y6]	815.3^{+}	30.0	
**IS**	GWGG	376.1^{++}	W [y3]	319.1^{+}	12.7	8.7
			G [y2]	133.0^{+}	21.7	
			G [y1]	76.0^{+}	21.7	
			W [b2]	244.1^{+}	12.7	
			G [b3]	301.1^{+}	6.7	

n.d. = not detected; Q1 = Precursor mass; Q3 = Transition mass; CE = Collision energy

Table S2 The optimized conditions for the multiple reaction monitoring (MRM) for the analysis of wheat samples. Q1 = Precursor mass; Q3 = Transition mass; CE = Collision energy

Protein	Quantifier peptides	Fragment	Q1-Mass	Q3-Mass	CE [eV]	Retention time [min]
P01083	K.VSALTGCR.A	A[y6]	432.2^{++}	677.3^{+}	14.4	8.0
		L[y5]		606.3^{+}	14.4	
		T[y4]		493.2^{+}	14.4	
P01083	K.VPIPNPSGDR.A	P[y9]	526.3^{++}	952.5^{+}	23.3	9.1
		P[y7]		742.3^{+}	23.3	
		P[y5]		531.3^{+}	26.3	
P17314	R.TNLLPHCR.D	L[y6]	505.8^{++}	795.4^{+}	19.7	8.6
		P[y4]		569.3^{+}	19.7	
		L[b3]		329.2^{+}	19.7	
P16850	R.SDPNSSVLK.D	P[y7]	473.7^{++}	744.4^{+}	15.7	8.1
		S[y5]		533.3^{+}	21.7	
P01084/85	K.LTAASITAVCR.L	T[y10]	581.8^{++}	1049.5^{+}	25.0	10.2
		S[y9]		806.4^{+}	22.0	
		T[y5]		606.3^{+}	25.0	
P15851	R.TSDPNSGVLK.D	P[y7]	509.3^{++}	714.4^{+}	19.8	8.1
		S[y5]		503.3^{+}	25.8	
		P[y7]		357.7^{++}	19.8	
P16159	R.YFMGPK.S	F[y5]	371.7^{++}	579.3^{+}	12.5	9.7
		M[y4]		432.2^{+}	12.5	
		G[y3]		301.2^{+}	12.5	
		P[y2]		244.2^{+}	12.5	
P16159	R.EVQMDFVR.I	Q[y6]	512.3^{++}	795.4^{+}	16.9	10.9
		M[y5]		667.3^{+}	16.9	
		D[y4]		536.3^{+}	13.9	
		F[y3]		421.3^{+}	25.9	
P81496-Q43723-Q43691	R.EQCVPGR.E	C[y5]	423.2^{++}	588.3^{+}	14.0	6.85
		V[y4]		428.3^{+}	14.0	
		P[y3]		329.2^{+}	14.0	
P93602	R.CEALR.V	E[y4]	324.7^{++}	488.3^{+}	14.1	6.95
		A[y3]		359.2^{+}	11.1	
		L[b4]		474.2^{+}	11.1	
P83207	R.ELAAISSNCR.C	A[y7]	560.8^{++}	807.4^{+}	18.4	8.9
		I[y6]		736.3^{+}	21.4	
		S[y5]		623.3^{+}	21.4	
		S[y4]		536.2^{+}	18.4	
Q4U199	K.LTAASITAVCK.L	T[y10]	567.8^{++}	1021.5^{+}	25.0	10.0
		S[y7]		778.4^{+}	22.0	
		T[y5]		578.3	25.0	
Q41540	R.NYVEEQACR.I	Y[y8]	608.3^{++}	1054.5^{+}	25.1	8.0
		V[y7]		891.4^{+}	19.1	
		E[y6]		792.3^{+}	19.1	
		E[y5]		663.3^{+}	19.1	
Q41540	R.IEMPGPPYLAK.Q	M[y9]	608.3^{++}	973.5^{+}	13.9	11.8
		P[y8]		842.5^{+}	28.9	
		G[y6]		745.4^{+}	19.9	
		P[y6]		688.4^{+}	13.9	
